# Causal relationship between schizophrenia and tachyarrhythmia: A Mendelian randomization study based on a European population

**DOI:** 10.1097/MD.0000000000042592

**Published:** 2025-10-24

**Authors:** Panpan Li, Zhenyu Yang, Mengzhu Chen, Jixin Li, Xiaohan Xiu, Weibo Zhong, Zheng Tang, Xihao Chen, Haohong Zheng, Xinyu Zhu, Zhigang Sun, Dandan Guo

**Affiliations:** a School of Medicine and Life Sciences, Zhangjiajie College, Zhangjiajie, China; b Heilongjiang University of Chinese Medicine, Harbin, China; c Xiyuan Hospital, China Academy of Chinese Medical Sciences, Beijing, China; d The First Affiliated Hospital of Chongqing Medical University, Chongqing, China; e College of Acupuncture and Massage, Shandong University of Traditional Chinese Medicine, Jinan, China; f The Second Affiliated Hospital of Heilongjiang University of Chinese Medicine, Harbin, China.

**Keywords:** atrial fibrillation, atrial flutter, Mendelian randomization, schizophrenia, supraventricular tachycardia, tachyarrhythmia

## Abstract

Given the emerging evidence suggesting a link between schizophrenia (SCZ) and tachyarrhythmias, this study utilized a bidirectional Mendelian randomization (MR) approach to explore the causal relationship between SCZ and several common tachyarrhythmias, including tachycardia, atrial flutter (AFL), atrial fibrillation (AF), and supraventricular tachycardia (SVT). Stringent selection criteria were applied to identify instrumental variables, followed by 5 MR analyses grounded in different hypotheses to evaluate the causal relationship between SCZ and tachycardia, SVT, and “AFL and AF.” Inverse variance weighted (IVW) was the primary MR analysis method used. Additionally, several sensitivity analyses were performed, including the MR pleiotropy residual sum and outlier global test, Cochran *Q* test, MR-Egger intercept test, leave-one-out sensitivity analysis, and bias risk assessment. The bidirectional MR analysis revealed that SCZ is a risk factor for both SVT and “AFL and AF.” Specifically, SCZ was found to significantly impact SVT [*P*_IVW_ = .038, odds ratio = 1.001, 95% confidence interval: 1.000–1.002] and “AFL and AF” (*P*_IVW_ = .005, odds ratio = 1.001, 95% confidence interval: 1.000–1.002). All studies were free of significant pleiotropy as well as genetic heterogeneity, the results of the studies were independent of individual genetic variant, and the selection of instrumental variables was unbiased. In a bidirectional MR analysis, we found that SCZ is a risk factor for SVT as well as “AFL and AF,” and that early intervention in patients with SCZ associated with tachyarrhythmias may be the key to improving their survival.

## 1. Introduction

With the prevalence of population aging problems and the increasing stress of life, arrhythmia has become an important public health problem worldwide, with high morbidity and mortality rates.^[1]^ Tachyarrhythmias are a group of disorders in which the pacing site emits electrical signals at an abnormally high frequency, repeatedly stimulating the heart over a period of time or causing ectopic cardiac beats, resulting in an increase in the heart’s rhythm, including tachycardia, atrial flutter (AFL), atrial fibrillation (AF), and supraventricular tachycardia (SVT).^[2]^ Tachyarrhythmias are usually rapid and complex, which not only aggravate myocardial oxygen consumption and induce myocardial ischemia, but also lead to heart failure (HF) and even acute myocardial infarction, bringing great pain to patients’ life and work. The worldwide prevalence of AF is about 2% to 4%, which is the most common type of arrhythmia in clinical practice, and its prevalence increases exponentially with age, bringing a serious economic burden to the society.^[3]^ AF refers to the loss of regular and orderly atrial electrical activity, which is replaced by fast and disorderly fibrillation waves, which deprives the atria of the effective systolic and diastolic functions, and ultimately affects the pumping function of the heart, increasing patients’ stroke, myocardial ischemia, and increasing patients’ risk of stroke, myocardial infarction, and even myocardial ischemia. This ultimately affects the pumping function of the heart and increases the risk of cardiovascular diseases such as stroke and myocardial infarction.^[4,5]^ AFL is a fast and regular atrial arrhythmia formed by refractory excitation in the atria, which is mostly paroxysmal and usually lasts for a few seconds, minutes, or hours.^[6]^ AFL is most commonly seen in patients with organic heart disease, and it is usually comorbid with AF, or it can be an isolated episode, but repeated episodes of AFL will eventually develop into permanent AF.^[7]^ SVT is a tachyarrhythmia due to lesions in the conduction system of the His bundle as well as above the His bundle, and patients tend to suffer from palpitations, dizziness, anxiety, chest tightness, and shortness of breath, and in severe cases, they may suffer from HF.^[8]^ It is most commonly seen in patients without organic heart disease, with a higher prevalence in women than in men, and is also known as paroxysmal SVT due to its sudden onset and cessation.^[9]^

The prevalence of schizophrenia (SCZ) is high among psychiatric disorders, and it is a relatively serious chronic mental illness, which is characterized by disturbances in mood, thinking and behavioral activities.^[10]^ Patients with SCZ mainly suffer from symptoms such as fantasy, depression, affective decompensation, social withdrawal and cognitive deficits, and in severe cases, they may also suffer from extreme violent tendencies and suicidal tendencies.^[11]^ The exact pathogenesis of SCZ is not well understood, and genetics, brain structure, pregnancy problems, and the influence of the surrounding environment may all contribute to its development.^[12]^ We speculate that when patients are under high stress and severe emotional fluctuations, it may lead to increased sympathetic excitability, which in turn causes rapid heartbeat and arrhythmia; and the occurrence of tachyarrhythmia may lead to increased anxiety, which promotes the development of SCZ. However, the results of existing observational studies are unreliable due to small sample sizes and the inability to exclude confounding factors and reverse causality.^[13–15]^ Conducting randomized controlled trials (RCTs) to establish a causal relationship between SCZ and tachyarrhythmia would require substantial resources and time and may raise ethical concerns, making it impractical for large-scale studies.

Mendelian randomization (MR) offers an efficient, cost-effective alternative to traditional RCTs, avoiding ethical issues and making it particularly suitable for large-scale causality screening. MR uses single nucleotide polymorphisms (SNPs) as instrumental variables (IVs) to explore the association between exposure and outcome, based on data from genome-wide association study (GWAS).^[16]^ According to Mendel second law, genetic variation from parent to offspring is randomly assigned, thus avoiding bias due to confounding factors in observational studies as well as the interference of reverse causality, and it is an effective method to reveal the effect of exposure on the results.^[17]^ For example, researchers have used GWAS data to identify mental illnesses that may worsen HF, providing valuable insights for treatment strategies.^[18]^ Similarly, bidirectional MR analysis has demonstrated that major depression increases the risk of coronary heart disease, suggesting that psychological counseling may be beneficial for coronary heart disease patients.^[19]^ Additionally, studies using GWAS data have confirmed that obesity shortens telomere length and accelerates epigenetic aging, offering direction for aging prevention.^[20]^ Inspired by the above studies, the present study will use a bidirectional MR study to reveal the causal relationship between SCZ and several common tachyarrhythmias (tachycardia, SVT, and “AFL and AF”), with a view to providing a basis for disease prevention and clinical treatment.

## 2. Methods

### 2.1. Study design

**Figure 1.**
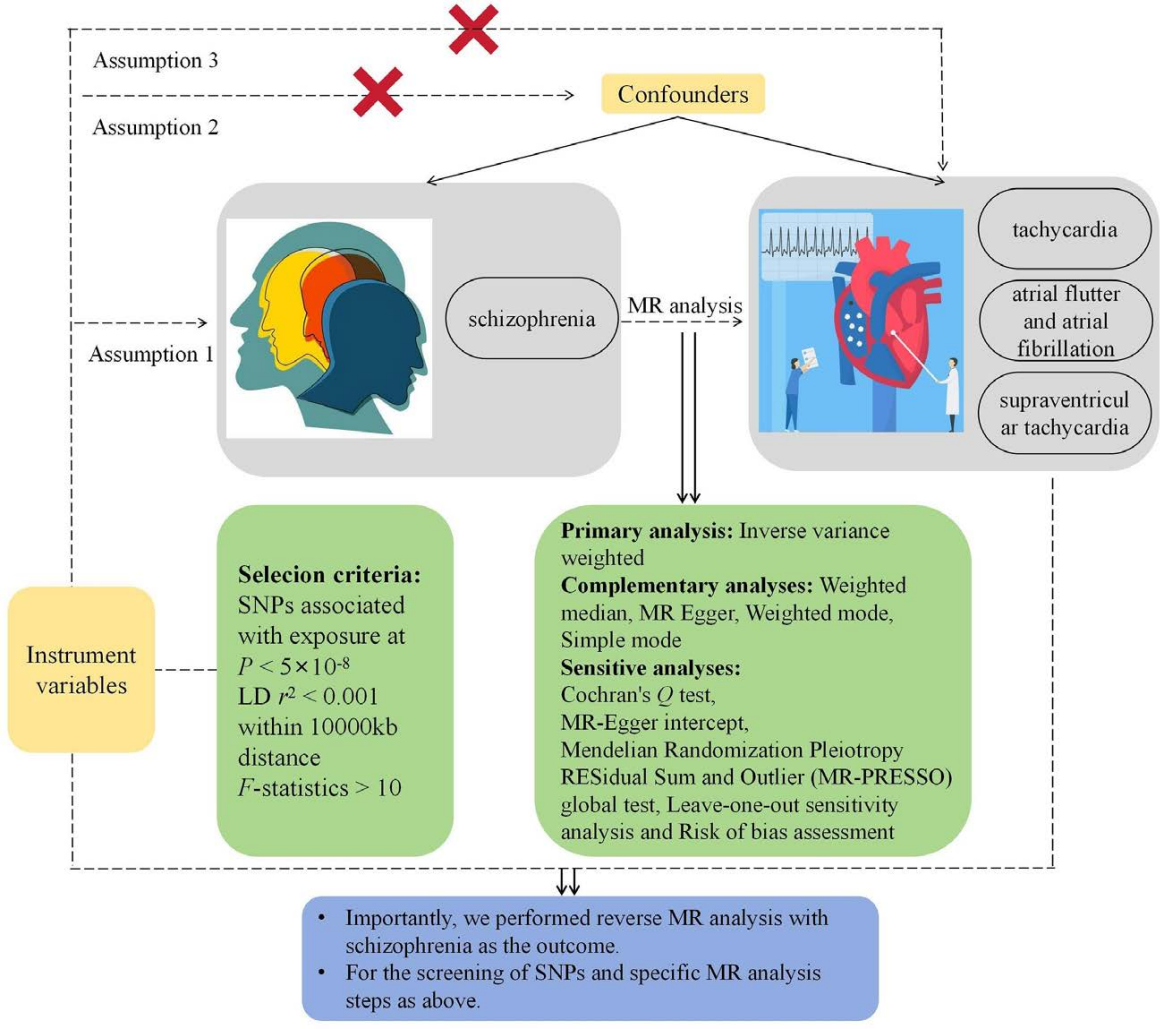
The basic idea map of this study.

The following 3 core assumptions must be followed in this MR study^[21]^: association assumption: genetic variants must be closely associated with the exposure factors; independence assumption: genetic variants should be independent of any confounding factors related to both exposure and outcome; and exclusion assumption: genetic variants should influence the outcome only through the exposure factors, without direct effects on the outcome. In this study, we used SCZ as an exposure factor and tachycardia, SVT, and “AFL and AF” as endpoints, and screened appropriate IVs for MR analysis. In addition, we also performed reverse MR analysis with the above tachyarrhythmias as exposure factors and SCZ as an outcome. The flow map of this study is shown in Figure 1.

### 2.2. Data source

The pedigree of this study was restricted to the European population to reduce bias due to demographic factors. Pooled data for the SCZ were obtained from IEU open GWAS (GWAS ID: ieu-b-5102, https://gwas.mrcieu.ac.uk/datasets/ieu-b-5102/). The study included 127,906 European individuals and was divided into a disease group (n = 52,017) and a control group (n = 75,889). The diagnostic strategy for SCZ was as follows: consensus between psychiatrists according to Diagnostic and Statistical Manual of Mental Disorders or International Classification of Diseases criteria; diagnostic interviews (e.g., structured psychiatric assessment); review of medical records or hospital registers; and review of the medical history of SCZ, hospital registers, and a mixed strategy using a combination of the previous methods. Pooled data for several tachyarrhythmias in this MR study were obtained from UK Biobank, involving a total of 463,010 European individuals, including tachycardia (n_case_ = 1005, n_control_ = 462,005, n_SNPs_ = 9851,867, GWAS ID: ukb-b-17309, https://gwas.mrcieu.ac.uk/datasets/ukb-b-17309/); SVT (n_case_ = 1306, n_control_ = 461,704, n_SNPs_ = 9851,867, GWAS ID: ukb-b-11748, https://gwas.mrcieu.ac.uk/datasets/ukb-b-11748/); “AFL and AF” (n_case_ = 6900, n_control_ = 456,110, n_SNPs_ = 9851,867, GWAS ID: ukb-b-6217, https://gwas.mrcieu.ac.uk/datasets/ukb-b-6217/). Diagnostic criteria for tachycardia: a heart rate of more than 100 beats per minute; exclusion of physiological tachycardia; and exclusion of other types of arrhythmia; diagnostic criteria for SVT: fulfillment of the electrocardiographic diagnostic criteria for SVT; a rapid and regular rhythm audible on cardiac auscultation; recent symptoms such as palpitations, dizziness, anxiety, chest tightness or shortness of breath; a past history of frequent episodes of SVT; and a mixed strategy using a combination of the previous methods; diagnostic criteria for AFL and AF: fulfillment of the electrocardiographic diagnostic criteria for either AFL or AF; an abnormal cardiac auscultation; a recent history of palpitations, vertigo, chest tightness, shortness of breath, malaise, or polydipsia; a past history of frequent episodes of AFL or frequent history of AF; and a mixed strategy combining the previous approaches. Only summary-level statistics were used in this study and all data were approved by the appropriate original ethical review boards; therefore, no additional ethical approvals were required.

### 2.3. Screening of IVs

In order to screen for eligible IVs, we developed strict screening criteria using the TwoSampleMR package (version 0.5.7) in the R.4.3.2 software. First, the genome-wide significance level was set to *P* < 5 × 10^-8^ and specified to be in the range of kilobase pairs (kb) = 10,000, with *r*^2^ < 0.001. This would filter out a large number of SNPs that were not closely associated with the exposure factors as well as those with linkage disequilibrium. Second, in order to prevent weak IVs from biasing the results, we calculated *F*-statistics and removed IVs with *F*-statistics < 10.^[22]^ Additionally, SNPs with palindromic structures were removed using the “harmonise_data” function to ensure consistency between exposure and outcome datasets.^[23]^ Finally, SNPs associated with confounding factors such as smoking, alcohol consumption, caffeine intake, physical inactivity, hypertension, obesity, and diabetes were excluded using the PhenoScanner V2.0 database. It is important to note that we make sure that the *P*_outcome_ >* P*_exposure_ for each SNP prior to performing MR analyses, which ensures that all IVs only influence the outcome through exposure, which also satisfies the exclusion assumption of MR.

### 2.4. Statistical analysis

#### 2.4.1. MR analysis methods

Five robust methods were employed for MR analysis, each based on different assumptions: inverse variance weighted (IVW), weighted median (WM), MR-Egger, weighted mode, and simple mode. IVW treats each genetic variant as an independent IV and estimates the common effect of multiple genetic variants on the disease while accounting for various confounding factors, making it the predominant method of analysis.^[24]^ MR-Egger uses the slope of weighted linear regression for causal estimation and is more reliable when there are numerous invalid IVs or when the findings may be polytomous.^[25]^ The WM method is applied when at least 50% of the IVs are assumed to be valid, providing a credible estimate even if nearly half of the IVs are invalid.^[26]^ Simple mode and weighted mode methods, being less statistically robust compared to the first 3, are used primarily as complementary approaches.^[27]^ In cases where different analytical methods yield different conclusions, the IVW method takes precedence. The results of these 5 MR analyses are primarily presented through scatter plots, where 5 diagonal lines represent the 5 different analytical methods. At a significance level of *P* < .05, an odds ratio (OR) > 1 indicates that exposure is a risk factor for the outcome, with the line slanting upwards; an OR < 1 indicates a protective factor, with the line slanting downwards. To increase the study’s confidence, we required that the OR values’ directionality across all 5 MR analysis methods be consistent. Additionally, a forest plot was created for the IVW, WM, and MR-Egger results, which includes the names of the 3 methods, *P*-values, OR values, and 95% confidence intervals (CIs) for the ORs.

#### 2.4.2. Sensitivity analysis

To further validate the results, several sensitivity analyses were conducted. First, horizontal pleiotropic outliers were identified and removed using the MR pleiotropy residual sum and outlier (MR-PRESSO) global test.^[28]^ For MR-PRESSO to provide accurate estimates, at least half of the IVs must be valid.^[29]^ A significant MR-PRESSO global test result (*P* < .05) indicates the presence of horizontal pleiotropic outliers, which need to be excluded; otherwise, no such outliers are present. Next, heterogeneity was assessed using Cochran *Q* test, and directional pleiotropy was evaluated using the MR-Egger intercept test.^[30]^ A significant result (*P* < .05) suggests the presence of heterogeneity or pleiotropy. If directional pleiotropy is detected, it implies that the MR analysis is unreliable. However, a small degree of heterogeneity is acceptable given the inherent differences between various SNPs. Additionally, a leave-one-out sensitivity analysis was performed, where the stability of the results was tested by sequentially removing each SNP from the exposure dataset. If the removal of a particular SNP significantly alters the results, it indicates that the MR analysis is primarily influenced by that SNP.^[31]^ Finally, a funnel plot was generated to assess the overall risk of bias. The horizontal axis of the funnel plot represents the effect size of the SNPs on the outcome, while the vertical axis indicates the reliability of the causal relationship. Each SNP is represented by a black dot, and a symmetric distribution of these dots around the central axis suggests that the MR analysis results are reliable and the selection of SNPs is unbiased.

#### 2.4.3. Reverse MR analysis

The primary focus of this study was to examine the effect of SCZ on various tachyarrhythmias. The reverse MR analysis served as a complementary study to the main findings. In the reverse analysis, SCZ was used as the outcome variable, and the aforementioned tachyarrhythmias were considered as exposure variables, followed by MR analysis. The same IVs screening criteria and analytical methods as described above were applied in this reverse MR analysis.

## 3. Results

### 3.1. Results of IVs screening

After rigorous screening, we obtained 158 SNPs that were strongly associated with SCZ and had no linkage disequilibrium (*r*^2^ < 0.001). As eligible IVs, these SNPs were within the physical distance threshold (10,000 kb) and had genome-wide significance (*P* < 5 × 10^-8^). For the MR analysis using tachycardia as the outcome, 55 SNPs were ultimately included. The *F*-statistics for these SNPs all exceeded 10 (range: 29.457–103.676), and the MR-PRESSO global test indicated the absence of horizontal pleiotropic outliers. When SVT was used as the outcome, 84 SNPs were included, with *F*-statistics similarly above 10 (range: 29.457–103.676) and no horizontal pleiotropic outliers detected by the MR-PRESSO global test. For the outcome of “AFL and AF,” 3 horizontal pleiotropic outliers (rs12833624, rs2078266, and rs7112616) were detected by the MR-PRESSO global test. After removing these outliers, 124 SNPs remained for the MR analysis, with *F*-statistics all above 10 (range: 29.457–175.278). Detailed results are provided in Tables S1 to S3, Supplemental Digital Content, https://links.lww.com/MD/Q441.

### 3.2. Results of the statistical analysis

**Figure 2.**
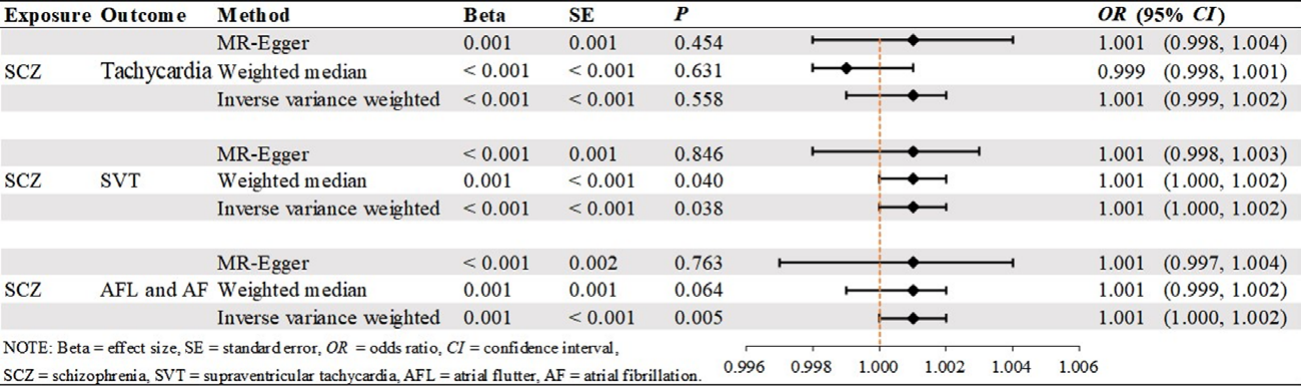
Forest map of key findings.

**Figure 3.**
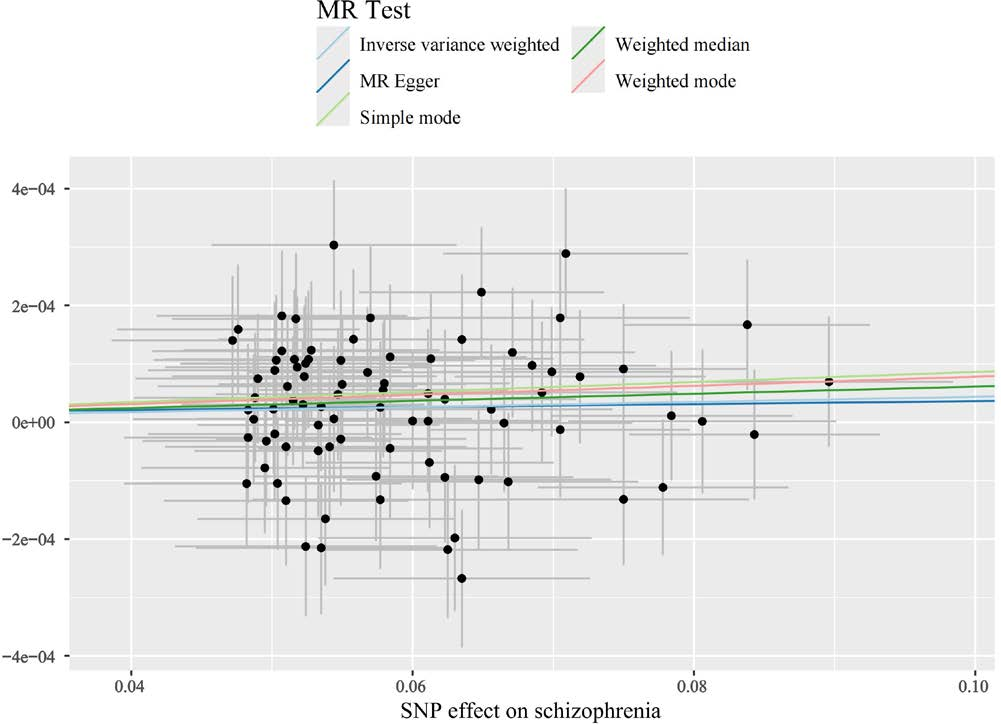
Scatter plot when SVT is the ending. SVT = supraventricular tachycardia.

**Figure 4.**
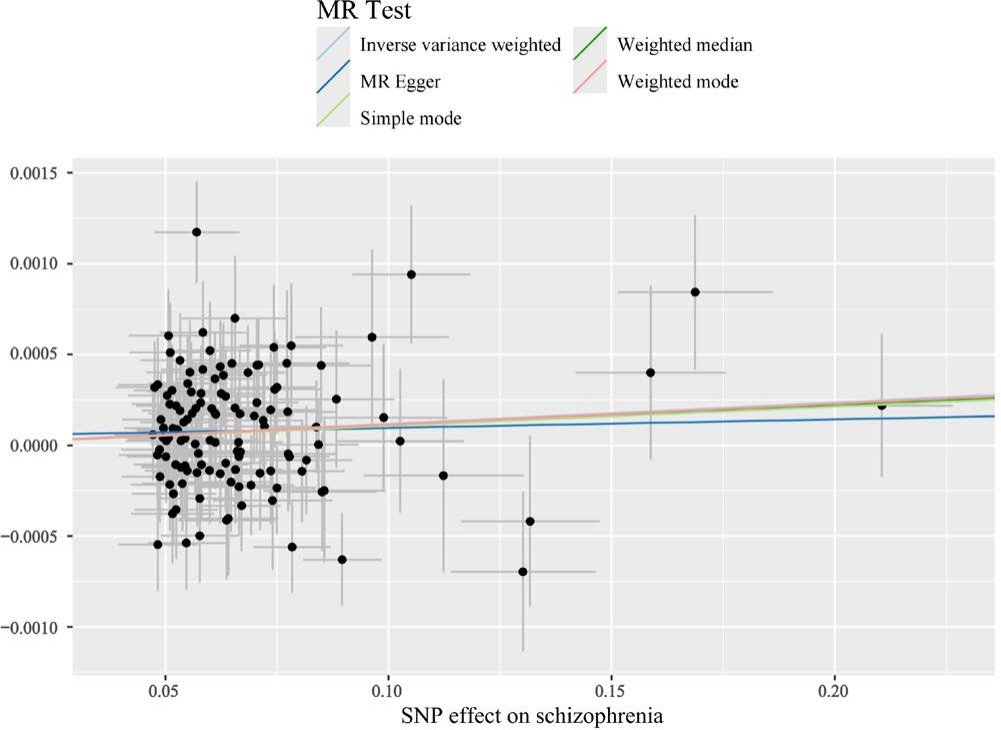
Scatter plot when “AFL and AF” is the ending. AF = atrial fibrillation, AFL = atrial flutter.

**Figure 5.**
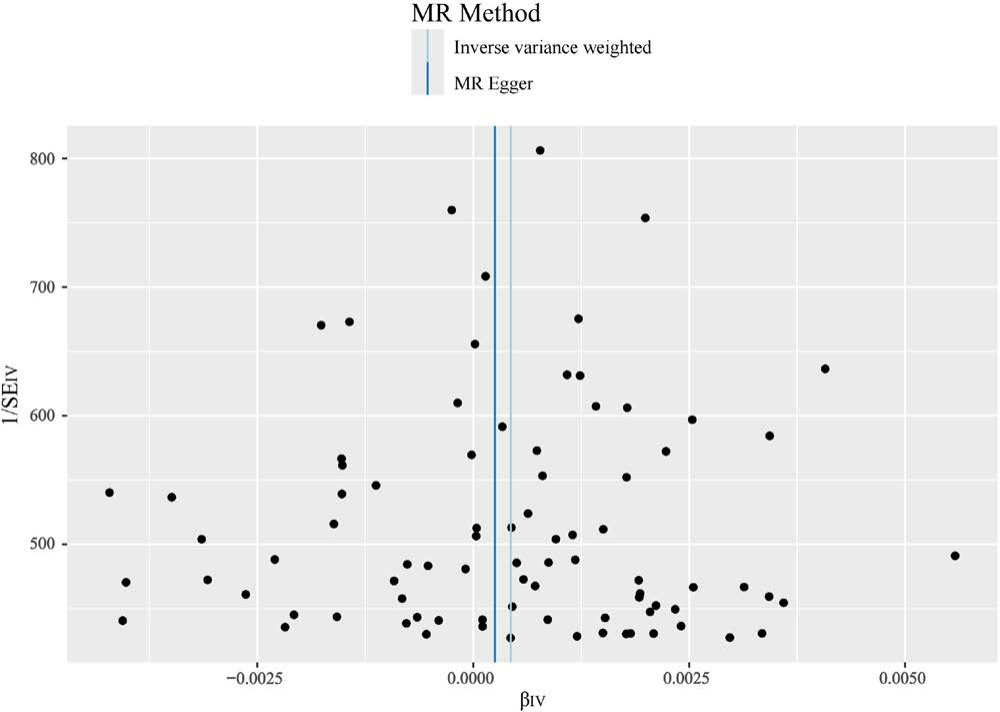
Funnel plot when SVT is the ending. SVT = supraventricular tachycardia.

**Figure 6.**
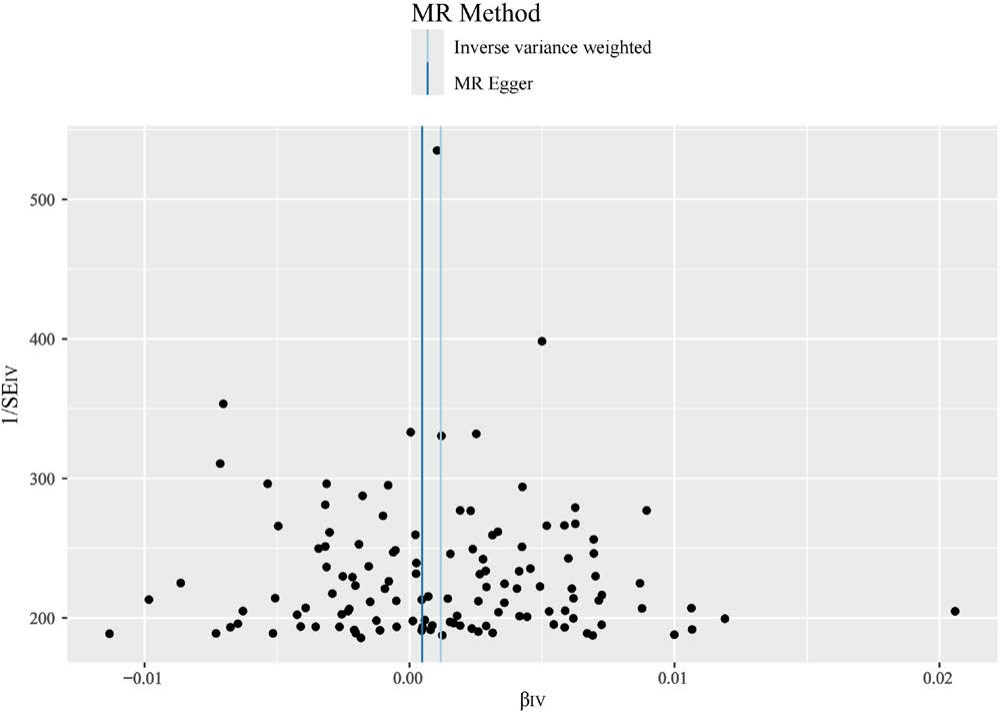
Funnel plot when “AFL and AF” is the ending. AF = atrial fibrillation, AFL = atrial flutter.

The main results of this study are shown in Figure 2, in which the effect of SCZ on tachycardia was not statistically significant when it was the outcome. SCZ was found to be a statistically significant risk factor for both SVT and “AFL and AF” with OR values >1 across all 5 MR analyses, as shown in Figures 3 and 4. Table 1 presents the results of the MR-Egger intercept test, which suggested a low likelihood of directional pleiotropy when SVT was the outcome. Similarly, Cochran *Q* test indicated a low probability of genetic pleiotropy when SVT was the outcome. When “AFL and AF” was the outcome, the MR-Egger intercept test also indicated a low likelihood of directional pleiotropy, although Cochran *Q* test suggested the presence of a small degree of genetic heterogeneity. Leave-one-out sensitivity analyses demonstrated that removing any single SNP did not significantly bias the results, as shown in Figures S1 and S2, Supplemental Digital Content, https://links.lww.com/MD/Q442. Funnel plots in Figures 5 and 6 show a relatively symmetrical distribution of SNPs, indicating that the MR analysis results are reliable and the selection of SNPs is unbiased. In the reverse MR analysis, when tachycardia and SVT were considered as exposure factors, no SNPs were identified for further analysis. However, when “AFL and AF” were used as exposure factors and SCZ as the outcome, a total of 13 SNPs were selected for subsequent analysis. Nevertheless, all MR analysis methods yielded *P*-values >.05, indicating that there was no statistically significant causal relationship between these exposure factors and SCZ. In other words, none of these types of tachyarrhythmias were identified as influencing factors for SCZ.

**Table 1:** Heterogeneity and multiplicity results table.

Exposure	Outcome	Cochran *Q* test		MR-Egger intercept test	
		MR-Egger	IVW	Egger_intercept	*P*
SCZ	SVT	*Q* = 81.197	*Q* = 81.218	<0.001	.884
		*P* = .504	*P* = .535		
SCZ	AFL and AF	*Q* = 149.553	*Q* = 149.823	<0.001	.639
		*P* = .046	*P* = .050		

AF = atrial fibrillation, AFL = atrial flutter, IVW = inverse variance weighted, MR = Mendelian randomization, SCZ = schizophrenia, SVT = supraventricular tachycardia.

## 4. Discussion

This study systematically explored the causal relationship between SCZ and 3 types of tachyarrhythmias using large, publicly available data from the IEU Open GWAS and UK Biobank databases. Utilizing SNPs as IVs, SCZ was identified as a risk factor for both SVT and “AFL and AF,” as evidenced by significant findings in IVW analyses (*P*_IVW_ < .05) and consistent ORs across 5 MR methods. Specifically, SCZ had a statistically significant impact on SVT (*P*_IVW_ = .038, OR = 1.001, 95% CI: 1.000–1.002) and “AFL and AF” (*P*_IVW_ = .005, OR = 1.001, 95% CI: 1.000–1.002).

The prevalence and lethality of SCZ are increasing yearly, and there is growing evidence suggesting that SCZ patients are at higher risk for cardiovascular diseases, with comorbidities being a key contributor to mortality in this population.^[32,33]^ Two primary factors contribute to the development of tachyarrhythmias: pathological substrates and disturbances in electrophysiology. An inflammatory response is closely associated with AF, AFL, and SVT, leading to atrial fibrosis, irregular hypertrophy, and even apoptosis of cardiomyocytes, which in turn creates pathological substrates for arrhythmia formation.^[34–36]^ Previous studies support this link between inflammation and arrhythmias. MA et al found that various inflammatory factors were positively correlated with AF risk in an MR analysis of a large GWAS database.^[37]^ MITTL et al reported that the prevalence of AF was associated with elevated levels of C-reactive protein (CRP), platelets, and neutrophils in a retrospective study.^[38]^ A prospective study showed that CRP and interleukin-6 levels were higher in patients with AFL and significantly reduced after radiofrequency ablation.^[39]^ Another study found that CRP levels were significantly higher in SVT patients compared to healthy controls, indicating a close relationship between the immune-inflammatory response and SVT development.^[40]^ SCZ, a complex disorder, is also characterized by chronic inflammation.^[41]^ Inflammatory mediators in SCZ may disrupt neurotransmitter function, leading to mood, cognition, and behavior disturbances. Mental disorders, including SCZ, have been associated with elevated CRP levels, which can exacerbate the production of pathological substrates contributing to arrhythmias.^[42,43]^ Persistent psychiatric disorders may also promote increased sympathetic excitability, activating the renin-angiotensin-aldosterone system and leading to disturbances in cardiac electrophysiology, which may precipitate tachyarrhythmias.^[44]^ In addition, elevated angiotensin II levels lead to atrial fibrosis, and atrial enlargement due to atrial fibrosis is strongly associated with the development of tachyarrhythmias.^[45,46]^ Finally, psychiatric disorders are known to reduce heart rate variability (HRV), promoting autonomic nervous system dysfunction and increasing the incidence of tachyarrhythmias such as AF.^[47]^ This is primarily due to fluctuations in autonomic tone or increased sympathetic activity caused by autonomic imbalance.^[48]^ ALVARES et al demonstrated through a meta-analysis that patients with psychiatric disorders, including SCZ, had lower HRV levels compared to healthy controls.^[49]^ AHN et al found that SCZ patients had a higher risk of tachyarrhythmias compared to other psychiatric patients, likely due to the lower HRV observed in SCZ.^[13]^ Although our study did not reveal a causal relationship between SCZ and tachycardia, the link between inflammatory responses and tachycardia has been reported previously.^[50]^ Future studies using larger GWAS databases may uncover more significant results. In conclusion, it is crucial to monitor cardiac rhythm in SCZ patients to prevent arrhythmias. Systematic management of comorbidities in SCZ patients is key to improving their survival.

Our study has several strengths. First, MR analysis is a rapid and efficient method for assessing causality, avoiding the limitations of observational studies that cannot fully control for confounding factors and reverse causality. MR analysis is also more cost-effective and efficient than traditional RCTs. Second, we used large-scale GWAS databases and applied strict criteria for IVs screening, enhancing the credibility of our findings. Third, reverse MR analyses showed minimal effects of tachycardia, “AFL and AF” and SVT on SCZ, which strengthens the robustness of our results. This underscores the importance of monitoring heart rhythm changes in SCZ patients to prevent arrhythmias. Lastly, by restricting the study population to European descent, we minimized demographic bias.

However, our study also has limitations. First, we did not account for the use of psychiatric medications, which can increase the risk of tachyarrhythmias.^[51]^ Psychotropic drugs may cause autonomic dysregulation or fluctuations in autonomic tone, affecting the heart’s electrophysiological balance and contributing to arrhythmias.^[52,53]^ Second, the data on tachyarrhythmias did not differentiate between types of AF (e.g., paroxysmal, persistent, permanent), which may have different etiologies. Additionally, the GWAS databases encompass diverse age, gender, and disease severity groups, precluding stratified analyses. Third, our study primarily included European populations, limiting the generalizability of the findings to other populations, which requires further validation through more reliable evidence. Forth, estimating the degree of overlap between exposure and outcome samples remains challenging. Finally, when several tachyarrhythmias were used as exposures, no positive results were obtained, which may be related to the sample size of the database, and larger sample sizes are still needed for future validation.

## 5. Conclusion

In conclusion, our bidirectional MR analysis, underpinned by a rigorous study protocol, evaluated the correlation between SCZ and 3 common tachyarrhythmias. The results suggest that SCZ is a risk factor for SVT and “AFL and AF” Early intervention in SCZ patients at risk for tachyarrhythmias may be crucial for improving survival outcomes.

## Acknowledgments

We thank Trubetskoy V et al for shared data on schizophrenia, and similarly we thank Ben Elsworth et al for shared data on tachyarrhythmias.

## Author contributions

**Conceptualization:** Zheng Tang.

**Data curation:** Weibo Zhong.

**Funding acquisition:** Panpan Li, Dandan Guo.

**Investigation:** Xinyu Zhu, Zhigang Sun.

**Methodology:** Xiaohan Xiu.

**Project administration:** Dandan Guo.

**Resources:** Mengzhu Chen.

**Software:** Zhenyu Yang.

**Supervision:** Dandan Guo.

**Validation:** Jixin Li.

**Visualization:** Panpan Li, Zhenyu Yang.

**Writing – original draft:** Zhenyu Yang, Mengzhu Chen.

**Writing – review & editing:** Panpan Li, Jixin Li, Xiaohan Xiu, Weibo Zhong, Zheng Tang, Xihao Chen, Haohong Zheng, Xinyu Zhu, Zhigang Sun, Dandan Guo.

## Supplementary Material


