# Supplementary material for: Causal relationship between schizophrenia and tachyarrhythmia: A Mendelian randomization study based on a European population
Source: Medicine (Baltimore). 2025 Oct 24;104(43):e42592. doi: 10.1097/MD.0000000000042592 (PMC12558327; doi:10.1097/MD.0000000000042592)
Supplement: Supplementary file 2 [file medi-104-e42592-s002.docx]

Figure S1 Leave-one-out sensitivity analysis plot when SVT is the ending;





Figure S2 Leave-one-out sensitivity analysis plot when AFL and AF is the ending; Table S1 SNPs finally included in this MR analysis when tachycardia was used as the outcome; Table S2 SNPs finally included in this MR analysis when supraventricular tachycardia was used as the outcome; Table S3 SNPs finally included in this MR analysis when "atrial flutter and atrial fibrillation" was used as the outcome.
